# Comparison of Analgesia Methods Through a Web Platform in Patients Undergoing Thoracic Surgery: Pilot Design, Implementation, and Validation Study

**DOI:** 10.2196/56674

**Published:** 2024-10-08

**Authors:** Rosella Trò, Angelica Orecchia, Nicola Disma, Paolo Uva, Roberto Cavanna, Nicolò Zanardi, Michele Torre, Marco Massimo Fato

**Affiliations:** 1 Department of Informatics, Bioengineering, Robotics and System Engineering University of Genoa Genoa Italy; 2 Unit for Research in Anaesthesia Istituto di Ricovero e Cura a Carattere Scientifico Giannina Gaslini Genoa Italy; 3 Clinical Bioinformatics Istituto di Ricovero e Cura a Carattere Scientifico Giannina Gaslini Genoa Italy; 4 Pediatric Thoracic and Airway Surgery Unit Istituto di Ricovero e Cura a Carattere Scientifico Giannina Gaslini Genoa Italy; 5 Department of Neuroscience, Rehabilitation, Ophthalmology, Genetics, Maternal and Child Health University of Genoa Genoa Italy

**Keywords:** pectus excavatum, pain assessment, web platform, health care informatics

## Abstract

**Background:**

Pain management is a vital and essential part of postoperative pectus excavatum (PE) care. Given the lack of an international consensus on guidelines for postoperative handling and evaluation, further research is necessary to compare the efficacy of existing pain management methods regarding pain relief, side effects, and long-term outcomes. In this context, the use of eHealth solutions for data mining can enhance data collection efficiency, reduce errors, and improve patient engagement. However, these digital health care frameworks are currently underused in the context of pain management for PE.

**Objective:**

This research is part of the broader Cryoanalgesia for Pain Management After Pectus Excavatum Repair (COPPER) study conducted by Giannina Gaslini Children’s Hospital to address postoperative pain and recovery in PE patients treated with either standard thoracic epidural analgesia or cryoanalgesia, which is considered its innovative alternative approach. Specifically, this work is aimed at introducing a valuable tool for a comprehensive and quantitative comparison of the 2 analgesia strategies. The tool is a web and mobile app designed to facilitate data collection, management, and analysis of clinical data for pain assessment.

**Methods:**

The adopted approach involves a careful design based on clinician input, resulting in an intuitive app structure with 3 main screens. Digital surveys are borrowed from paper surveys, including medical history and preoperative, postoperative, and follow-up evaluations. XTENS 2.0 was used to manage the data, and Ionic facilitated cross-platform app development, ensuring secure and adaptable data handling.

**Results:**

Preliminary analysis on a pilot cohort of 72 patients (36 treated with standard therapy and 36 treated with cryoanalgesia) indicated successful patient enrollment and balanced representation across treatment groups and genders. Notably, hospital stay was significantly shorter with cryoanalgesia than with standard therapy (Mann-Whitney-Wilcoxon 2-sided test with Bonferroni correction; *P*<.001; *U* statistic=287.5), validating its treatment efficacy.

**Conclusions:**

This work is a step toward modernizing health care through digital transformation and patient-centered models. The app shows promise in streamlined data collection and patient engagement, although improvements in multilingual support, data validation, and incentivization of questionnaire completion are warranted. Overall, this study highlights the potential of digital health solutions in revolutionizing health care practices, fostering patient involvement, and improving care quality.

## Introduction

### Background

Pectus excavatum (PE) is by far the most common congenital chest wall deformity, which is characterized by the inward sinking of the sternum and adjacent costal cartilages, resulting in a concave depression in the chest [1,2]. This condition is usually diagnosed in childhood or early adolescence and may cause respiratory and cardiac problems if left untreated [3,4]. Currently, the most adopted surgical intervention is the Nuss procedure [5], also known as minimally invasive repair of PE, involving the positioning of a curved metal bar under the sternum to ensure its stabilization. However, despite the minimally invasive approach, patients often report postoperative pain in the affected area, which can persist long after the intervention and affect the resumption of daily activities. Pain management is thus a critical component of postoperative PE care, despite the overall lower incidence of chronic postoperative pain in the pediatric population than in the adult population [6].

In this respect, standard thoracic epidural therapy is considered the gold standard for the management of postoperative pain in PE, in addition to many other surgical procedures [7-9]. This technique involves inserting a catheter into the epidural space of the thoracic region to administer local anesthetics or opioids. Although it is highly effective in providing short-term pain relief, the pain often returns at catheter removal and persists even after discharge from the hospital.

To overcome this important issue, intercostal nerve cryoablation, namely cryoanalgesia, has recently emerged as an alternative pain relief technique [7,10,11]. This procedure involves congealing the nerve fibers around the surgical site using a specialized probe that delivers extremely cold temperatures. By disrupting the transmission of pain signals from the site to the brain, this technique has shown promising results in managing postoperative pain in various surgical procedures [12] and has been associated with substantially reduced postoperative length of stay and narcotic usage after Nuss surgery compared to thoracic epidural analgesia [7,13-15], though no significant differences in pain scores were reported between the 2 groups [16].

Nevertheless, studies on concerns about long-term neuropathy after axon regeneration have been carried out in the adult population [17,18], unlike in the pediatric population [19].

Given the lack of international consensus on guidelines for postoperative management and evaluation, further investigation is therefore needed in this direction to compare the efficacy of the 2 pain management methods and determine which method is superior in terms of pain relief, side effects, and long-term outcomes [20].

In an attempt to bridge this gap, Giannina Gaslini Children’s Hospital has proposed a randomized controlled trial (RCT) named Cryoanalgesia for Pain Management After Pectus Excavatum Repair (COPPER) registered at ClinicalTrials.gov, with the initial release date of September 20, 2021 (NCT05201820) [21]. Its aim is to monitor postoperative pain and return to normal daily activities in patients undergoing PE repair surgery and treated with either thoracic epidural analgesia or cryoanalgesia procedures. Specifically, the primary objective of the survey is to determine whether cryoanalgesia improves standard analgesic therapy after repair by means of specific clinical scores measuring life quality, pain intensity, side effects, and hospitalization time.

The results gathered from the COPPER study will be the object of a primary clinical paper centered on a robust comparison of the 2 analgesia strategies in terms of patient outcomes. Conversely, this work focuses on the first crucial step within such an ambitious project, which involves the development of a web and mobile app for gathering data from pain assessment questionnaires to be filled out by patients at regular intervals during convalescence from the repair surgery. Resorting to a mobile health (mHealth) app to fulfill this task ensures a systematic and thorough collection of the needed data, allowing, in turn, optimal pain management after the Nuss procedure, with undisputed benefits to not only the patient but also the hospital (eg, early discharge, decrease in the risk of hospital-acquired infection, and optimal space utilization within the hospital).

### Prior Work

To the best of our knowledge, in the context of PE, few to no studies have focused on web apps specifically conceived for handling the evaluation of pain in surgery candidates. 

A mobile app has been developed [22], but it is more focused on tracking general progress resulting from PE treatment rather than postoperative pain management. Another pioneering study deals with the implementation of an enhanced recovery pathway for minimally invasive pectus surgery using eHealth technology [23]. It is based on a web-based platform for psychological screening and telemonitoring for follow-up at home, thus paving the way for underscoring the potential of eHealth technologies in improving the management of postoperative pain and overall recovery in pediatric patients undergoing pectus surgery. However, a small sample size (29 patients), web surveys mainly limited to psychological screening, short-term follow-up (10 weeks), and a standard analgesia approach (preemptive epidural) underline its preliminary nature and limitations. 

### Goal of the Study

Our study involves the first digital app specifically designed for gathering, collecting, and handling data, thus allowing an efficient comparison between gold standard epidural analgesia and cryoablation methodologies, which will, in turn, improve the postoperative care of PE patients.

In this methodological paper, we will focus on illustrating the procedure of the design, development, and validation of such a web-based platform, hinting only briefly at the clinical implications inferred from the data collected so far.

## Methods

### Summary

The methodological pipeline has been conceived to design a web app adaptable to mobile devices for the translation of paper questionnaires, compilation of digital questionnaires, and storage of the data entered by the patients belonging to both groups. These online questionnaires must be filled out at regular intervals set by clinical practice with the purpose of collecting data about the patient’s pain and overall quality of life following surgery.

### Paper Pain Assessment Questionnaires

Paper forms have been used as a starting point for the conceptualization and development of digital forms to be compiled via the app. Their structure, also maintained in the online counterpart, can be subdivided into postoperative and follow-up surveys.

### Postoperative Pain Assessment Questionnaire

The postoperative questionnaire covers the first 13 days after surgery. If the patient is still hospitalized, the compilation is performed by or with the help of the hospital’s medical staff, while after discharge, the compilation is performed independently via the app. The survey in paper format consists of 10 questions mainly regarding (1) the frequency, (2) duration, and (3) worst value of pain experienced during the day through the Numeric Pain Rating Scale (NRS) [10], and (4) the self-perceived level of difficulty in performing daily activities measured by the Youth Acute Pain Function Ability Questionnaire (YAPFAQ) [11]. This questionnaire consists of a basic question “How difficult is it for you to do these things today?” and the patient rates the level of difficulty in performing each activity during the day using a 5-point scale (0=“easy,” 1=“somewhat strenuous,” 2=“rather strenuous,” 3=“very strenuous,” and 4=“extremely strenuous”). In total, the questionnaire consists of 12 questions. The total score ranges from 0 to 48 for all items present, with higher scores indicating greater difficulty in performing functional activities. Some of the questions belonging to the postoperative form, which are related to the dose and timing of analgesia (eg, morphine intake), must be answered only during the hospital stay, and they are automatically locked in the mobile app after insertion. A sample paper questionnaire is provided in Multimedia Appendix 1, and specific details about the YAPFAQ are reported in Multimedia Appendix 2.

### Follow-Up Pain Assessment Questionnaire

The follow-up questionnaire starts from the 14th day on a weekly basis for the first 2 weeks and then continues monthly until the 6th month after surgery. The questions regarding NRS, frequency, and duration of pain inherit the same structure as in the postoperative survey, while more specific questions are added about the patient’s quality of life through the YAPFAQ, Pediatric Quality of Life Inventory (PedsQL) [12], and Child Activity Limitations Interview-9 (CALI9) [13]. Specifically, the PedsQL is a valid, practical, brief, standardized, generic self-report assessment tool for measuring quality of health–related life in pediatric and adolescent patients. By integrating both generic baseline scales and disease-specific modules into a single measurement system, the PedsQL is applicable to both healthy and chronically ill populations. It contains 23 items overall, spanning the areas of physical operation (8 items), emotional functioning (5 items), social functioning (5 items), and school functioning (5 items). Each item is rated on a 5-point scale from 0 to 4 (0=“never a problem,” 1=“almost never a problem,” 2=“sometimes a problem,” 3=“often a problem,” and 4=“almost always a problem”). The scores for each question are summed without weighting of items, with a higher score corresponding to worse symptoms. Next, the scores are transformed linearly into a 0-100 scale (0=100, 1=75, 2=50, 3=25, and 4=0) in which a higher value indicates a better condition. The calculation of the total score is possible only if at least half of the available questions are filled in. On the other hand, the CALI9 is a measure to assess and monitor pain-related activity limitations in children and school-age adolescents with recurrent and chronic pain, and it includes 9 items. Participants rate the difficulty in completing each task on a 5-point rating scale, ranging from 0 (“easy”) to 4 (“extremely strenuous”), and a total score is obtained by summing the ratings for all 9 items (range 0-36), with a higher score indicating greater activity limitations due to pain. A sample paper questionnaire is presented in Multimedia Appendix 3, and specific details about the PedsQL are reported in Multimedia Appendix 4.

### App Architecture

**Figure 1:**
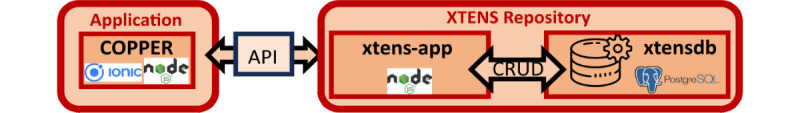
XTENS setup for the COPPER project. XTENS communicates with the app domain (COPPER) using REST. The web app compiled with Ionic calls the methods of the XTENS API. Once the patient has been authenticated (subjectLogin method of the API), the prospectus of the visits to be completed is shown (visitDataRetrieval method of the API), and once a visit is confirmed, the latter is saved to XTENSdb via the visitSaving method of the API. API: application programming interface; COPPER: Cryoanalgesia for Pain Management After Pectus Excavatum Repair; CRUD: create, read, update, and delete.

The designed app leverages pre-existing tools for collecting, querying, managing, and storing clinical data within the COPPER trial. Figure 1 displays an overview of the interaction among the main modules used for the development of such an app.

Moreover, we present our web survey reporting in accordance with the Checklist for Reporting Results of Internet E-Surveys (CHERRIES) [24] (Multimedia Appendix 5). The details of several key domains, including (1) the design and development process, (2) institutional review board approval and informed consent, (3) recruitment and data collection, (4) survey development and testing, (5) data privacy and security, (6) analytical methods, and (7) the presentation of results, are presented in this checklist (Multimedia Appendix 5).

### XTENS 2.0

For metadata collection and integration, we used XTENS 2.0 [25], an innovative data repository able to provide adaptive metadata management and configuration tools to maximize information sharing and understanding [26]. Being a JSON-based metadata repository specifically designed for biomedical sciences, it allows for flexible and extensive metadata support. This platform has been successfully used in different scenarios, ranging from neuroscience to biobanking and functional genomics, owing to its ability to cope with the heterogeneity inherent to biomedical data (eg, clinical records, biological specimens, imaging and genomic data, and different technology-associated formats). XTENS 2.0 is based on a JSON metadata model [27], which is, in turn, implemented using a JSON-compliant modular structure consisting of a web app running on a Node.js server, a PostgreSQL database, and a distributed file system based on the iRODS data grid software [28]. This allows fast, direct, and transparent access to data from the client side. After mandatory initial configuration and deployment, new *data types* can be created with little to no effort or specific technological expertise. Indeed, in XTENS, the users are given full control to create new *data types* and setups relative to security and authorization levels through an intuitive graphical interface. Further details can be obtained from the literature [29,30].

**Figure 2:**
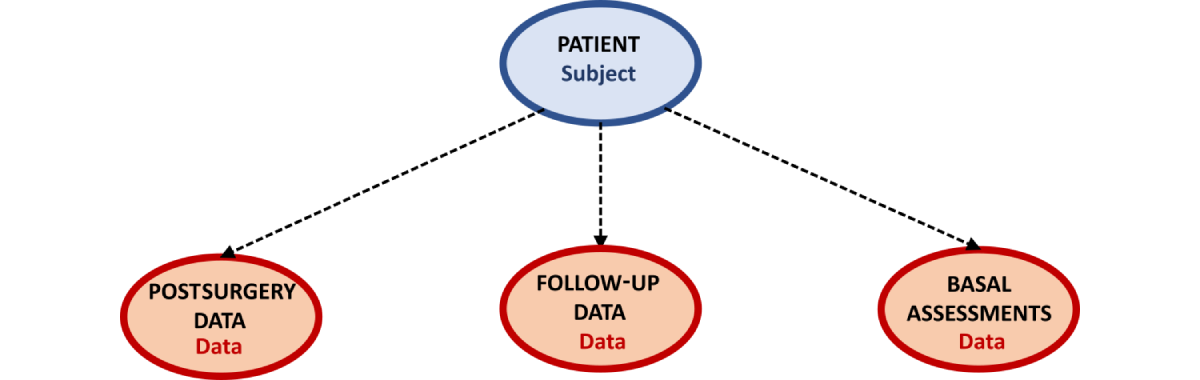
Outline of the XTENS data model in the use case of the COPPER study. Each “data” instance is characterized by its “data type,” and the “data type” schema provides the structure to build up the metadata JSON object. COPPER: Cryoanalgesia for Pain Management After Pectus Excavatum Repair.

Regarding our work, we have installed XTENS on a server (Centos 7) located at the University of Genoa. We thus created the data structure of the questionnaires exploiting XTENS hierarchical JSON schema based on *data type* objects, which characterize corresponding *data* instances. The following 4 different *data types* were created: 1 of *subject* for the definition of the patient and 3 of *data* for questionnaire data, which are children *data types* of *patient* data (Figure 2).

The *data type* of *subject*, called *patient*, aims to define the patient who has been enrolled in the study. It is about a specialized version of the *data* instance class, containing additional fields, methods, and relationships. To create a new patient, the first name, last name, and date of birth must be entered. Each new patient is automatically assigned an alphanumeric code consisting of a prefix and a number, which incrementally increases starting from 1 for the first patient created. This is the patient’s identification code for both accessing the app and searching related data via the XTENS platform. The following attributes have also been included to define the characteristics of the patient detected during enrollment by medical staff:

- Date of surgery
- Password: alphanumeric string that will be given to the patient upon discharge for access to the web app
- Group: indicates whether the patient underwent PE reconstruction with thoracic epidural or with cryoanalgesia
- Weight: subject’s weight expressed in kilograms at the time of enrollment in the study
- Height: subject’s height expressed in centimeters at the time of enrollment in the study
- Haller index: decimal number representing the most commonly used index of PE severity
- Index of correction: decimal number representing the most representative sternal depression index, expressed as a percentage
- Concomitant pathologies: free-text field
- Comments: free-text field
- Date of discharge
- Maximum inspired volume: integer number indicating the maximum volume of air that can be inhaled at one time, expressed in milliliters

There are 3 *data types* subordinate to the *patient data type*. The first subtype is *baseline assessments*. Each individual participating in the study was subject to a preoperative visit to determine the quality of life prior to chest wall reconstruction. Attributes were associated with this type of data, which did not correspond to a paper survey. These included (1) NRS: an integer number, to be chosen from a list of values between 0 and 10, representing the worst value of pain experienced on that day; (2) PedsQL: with individual attributes for the total obtained for each of the 4 sections (PedsQL 1, PedsQL 2, PedsQL 3, and PedsQL 4), along with the attribute containing the total score achieved for all questions present (PedsQL total score); (3) CALI9: integer number measuring the sum of the score obtained when filling out the CALI9 questionnaire; (4) YAPFAQ: integer number representing the sum of the score obtained during completion of the YAPFAQ; (5) American Society of Anesthesiologists (ASA) physical status classification: text string with a choice among 4 possible values for classifying the patient’s preintervention physical status; (6) Comments: free-text field in which medical staff can enter annotations taken during the patient interview. The data models for postoperative and follow-up data have been created in collaboration with clinicians to reproduce existing paper questionnaires.

The second subtype is *postoperative data*. The NRS and YAPFAQ have been structured in the same way as in the *baseline assessments*. The other attributes included (1) day: integer number between 1 and 13 selectable through a list of preset values representing the postoperative day; (2) pain frequency: integer number between 0 and 4 expressing how many times during the day of the compilation pain has been experienced; (3) pain duration: integer number between 0 and 4 expressing how long a patient has been feeling pain during the day; (4) morphine consumption: as for morphine intake at the hospital, it was decided to structure it through 2 different attributes (one for morphine consumption expressed in total milligrams and the other for the time of administration); (5) fixed analgesia hours: Boolean value indicating whether the subject has received fixed analgesia; (6) side effects: for the evaluation of side effects, an attribute of the Boolean type to report the overall presence of effects, 6 other Boolean attributes for the 5 most common side effects (nausea, vomiting, allodynia, hyperalgesia, and lower extremity paresthesia), and a free-text attribute for secondary side effects encountered have been instanced, with the structure allowing to search for all subjects who generally experienced at least one side effect or a particular side effect; (7) date of compilation: despite the presence of the attribute *day* in the same model, we decided to include the actual date of compilation since, together with physicians, we established to allow a range of 2 days for completion; (8) analgesia as needed: to indicate whether the subject has taken extra analgesia in addition to fixed analgesia, 2 attributes were created (one of the Boolean type to state whether the need was there or not [analgesia on need] and the other of the integer type to indicate how many extra doses per day were taken [analgesia doses at need]); (9) motor skills: Boolean-type attribute for the 4 skills to be assessed during the period following surgery (being able to sit up, holding hands behind the back of the neck while sitting, walking, and performing a squat); and (10) physiotherapy judgment: free textbox containing physiotherapist comments.

The third subtype is *follow-up data*. The follow-up *data type* has no further attributes than those already explained for *baseline assessments* and *postoperative data* and involves a combination of these. Specifically, the NRS, PedsQL, CALI9, YAPFAQ, and comment attributes are from *baseline assessments*, while the day, pain frequency, and pain duration attributes are from *postoperative data*.

### App Development

#### Ionic

Mobile app development was performed using Ionic 6.20.8 [31]. Ionic is an open-source user interface (UI) toolkit for building modern performant cross-platform hybrid apps for all major app stores and the mobile web from a single codebase. Ionic has been chosen for providing significant performance, usability, and feature improvements alongside support for all popular UI web frameworks like Angular, React, and Vue.js. Specifically, Ionic emulates native app UI guidelines and uses native software development kits (SDKs), bringing the UI standards and device features of native apps together with the full power and flexibility of the open web. It is indeed platform-independent. Developed apps can work on different mobile platforms like Android, iOS, and Windows without much effort, and this overcomes the platform-specific, time-consuming, and expensive requirements of native apps. This is in turn granted by the Apache Cordova or Capacitor mobile app development framework that helps developers to build a rapid mobile app deploying HTML5, CSS3, and JavaScript instead of using platform-specific application programming interfaces (APIs). Moreover, Ionic is designed to work and display beautifully on all current mobile devices and platforms owing to ready-made components, typography, and an extensible base theme that adapts to each possible platform.

Among all possible web app frameworks compatible with Ionic, we resorted to Angular (devkit core version 12.1.4). Furthermore, given the scale of our designed app, we opted for compiling it as a web app instead of compiling it for all mobile devices as usually performed with Ionic projects.

#### Angular

Angular (successor to AngularJS) [32] is a JavaScript framework for web app development. It works by scanning the HTML code of the current page, which has encapsulated additional custom attributes (eg, ng-controller), interpreting these attributes as directives (commands) to bind the input and output parts of the page to the model that is represented by standard JavaScript variables. Ionic uses a set of reusable and possibly expandable Angular directives for the graphical UI. The main advantage of using Angular is the implementation of bidirectional data-biding, which allows automatic synchronization of data from the UI (view) with JavaScript objects (model), thus improving testability and performance as well as making it easy to accomplish complex user interactions and updates to the UI in real-time. Indeed, most template systems support data-biding in only one direction, typically from the data model to the view. This means that the model data are combined with the HTML template to generate the view visible to the user. However, if the template is changed, the changes are not automatically reflected on the view. Similarly, if the user modifies the view, these adjustments are not directly synchronized with the data model. Synchronizing the view and model generally requires writing code that performs this function. Angular’s databinding, on the other hand, allows synchronization without the need to write any special code.

### App Security

With regard to secure storage and accessibility of patient data, all steps have been taken to ensure data protection: (1) both the web app and XTENS web app are password protected and can be accessed by admins with credentials; (2) all data transmission is performed via Secure Sockets Layer (SSL) encryption; and (3) only the web app can access the XTENS API due to IP check.

### Ethical Considerations

All procedures involving human participants were in accordance with the ethical standards of the institutional or national research committee and with the 1964 Helsinki Declaration and its later amendments or comparable ethical standards.

This study was approved by Comitato Etico Regionale Liguria, Italy (CER Liguria 278/2021 – DB ID 11421). Informed consent (no waiver) was required from each patient as per clinical trial requirements. The data were processed in compliance with the General Data Protection Regulation (GDPR) and deidentified during the scientific analysis. No compensation was provided to participants.

## Results

### Summary

In accordance with clinicians, we built the structure and graphics of the app following a minimal and intuitive design scheme to facilitate its use by patients and medical staff, as well as to try and avoid errors during compilation. The app consists of 3 main screens: *login*, *home*, and *questionnaires*.

The paper questionnaires, distributed in Italian, were translated into English for publication purposes. They have been included in Multimedia Appendices 1-4. The app was designed in Italian, without being structured to allow multilingualism, as the majority (if not all) of the patients were native speakers and within the Italian national health service. Nonetheless, a detailed explanation for each screen of the app has been provided.

### Mobile App Design

#### Login

**Figure 3:**
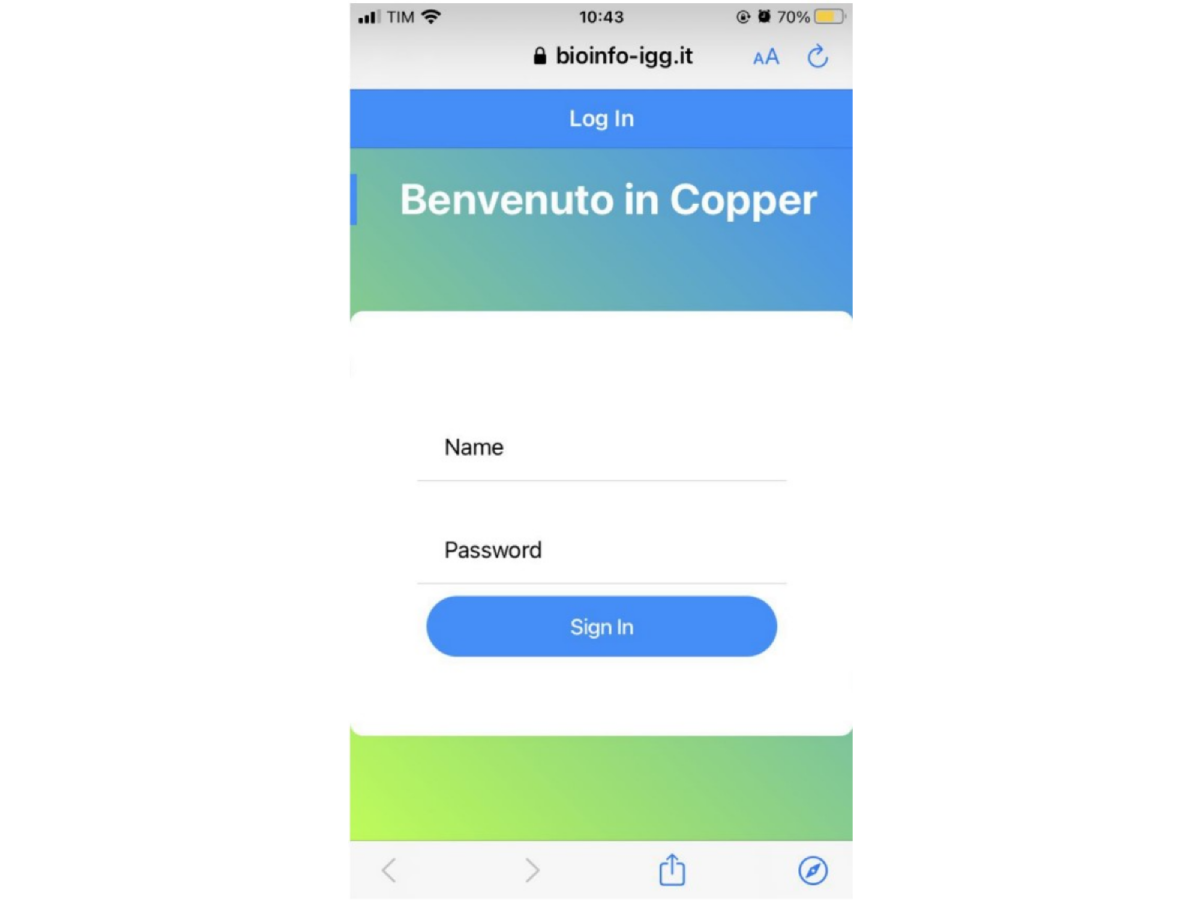
Login screen on an iOS device. It welcomes the patient and provides the opportunity to enter their credentials to access the app.

The *login* page has been designed so that when opening the app, a box appears for inserting the user’s name and password, which are directly provided by the medical staff on the last day of hospitalization, with a view to ease the autonomous compilation of surveys (Figure 3). Filling in is intuitive, responsive, and dynamic, with each box remaining highlighted in red until a string is entered. If the user accidentally clicks on the *sign in* button before entering the credentials, an error message appears, informing about the lack of one or more prerequisites for access. Once the credentials are entered and the button is clicked, the system sends a request to the XTENS server via the HTTPS protocol to search for a patient *subject* with these features. If authentication is unsuccessful, an error message is displayed indicating that the entered credentials are incorrect; otherwise, all the attributes of the patient *subject* are sent and thus saved locally on the device. This includes retrieving not only *patient* baseline data but also all received visits.

#### Home Page

**Figure 4:**
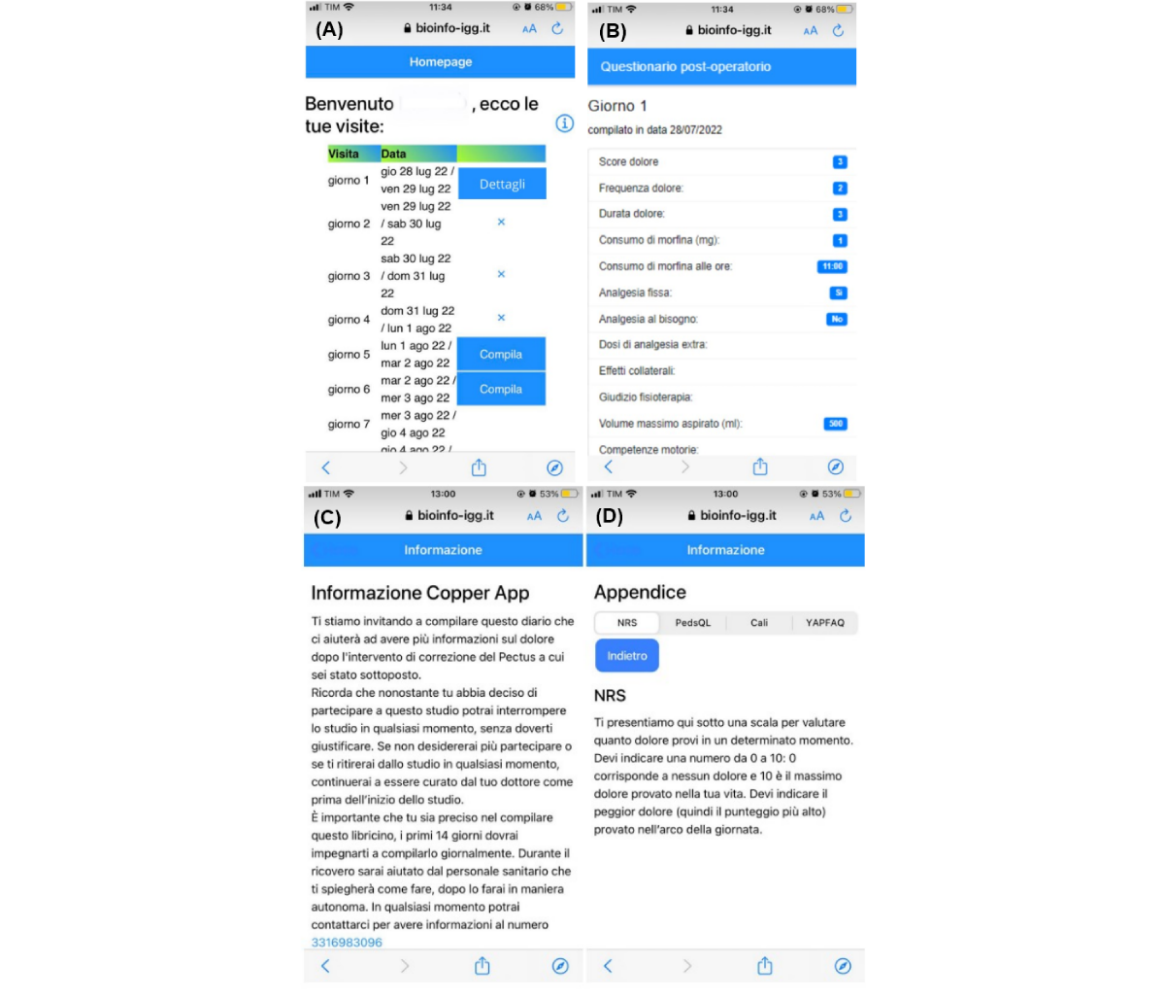
Home page screen on an iOS device. (A) The home screen displays an overview of all visits related to the specific patient, with date. If the examination has already been carried out, the user can access the details. If the examination is still to be carried out, the details can be obtained later. (B) The second screen contains all the attributes related to the specific questionnaire (eg, score, duration, and frequency of pain, etc). In this specific case, details about an exemplary (postoperative) questionnaire at day 1 are displayed. (C) The home page screen also contains an “information” section that acts as a guide for the patient to complete the questionnaires. When clicked, it links to a range of useful information about the COPPER study, including its purpose, patients’ rights and duties, filling-in instructions, and contact numbers for any problems. (D) In addition, it includes an “appendix” where it is explained how to answer and how the NRS, YAPFAQ, CALI9, and PedsQL questions are evaluated. CALI9: Child Activity Limitations Interview-9; COPPER: Cryoanalgesia for Pain Management After Pectus Excavatum Repair; NRS: Numeric Pain Rating Scale; PedsQL: Pediatric Quality of Life Inventory; YAPFAQ: Youth Acute Pain Function Ability Questionnaire.

Once the necessary data have been collected, the *home page* of the app appears to the user. This screen shows all the visits with the corresponding questionnaires that need to be completed. The date of expected questionnaire completion is automatically calculated since the date of the intervention is an attribute of the retrieved *patient* data. Theoretically, within the first 14 days, the patient should complete the questionnaires daily, but, in collaboration with the medical team, it was decided to leave a range of 2 days for completion. For the subsequent monthly questionnaires, a 5-day completion range was established. If the set time range is exceeded, an “*X*” is displayed, preventing the user from performing tasks for that questionnaire on the mobile app. Moreover, to avoid possible compilation errors, the questionnaire is not visible before the useful date of compilation. Through a control variable, the user can determine whether the questionnaire has already been completed (Figure 4A). In the first case, a *details* button appears in the relevant row, which allows the user to review the answers provided (Figure 4B). In the second case, a check is performed between the date at the time of *login* and the date range of the questionnaire. If they match, a *fill* button is displayed that redirects to the completion page. We have included an *appendix* for consultation at the top right of the *information* icon. If clicked, it brings the user to a page containing all the information about the COPPER study and contact numbers for any problems (Figure 4C), and illustrates specific details regarding the NRS, YAPFAQ, CALI9, and PedsQL surveys (Figure 4D).

### Online Pain Assessment Questionnaires

The pages pertaining to filling out the 2 types of questionnaires were developed with similar structures. After discussing with the medical team at Gaslini Hospital, we finally opted for multiple screens instead of presenting a single screen with all the questions. This layout allows easier compilation without mistakes or oversights.

Questions related to subquestionnaires, such as YAPFAQ or CALI9, have been placed on the same display page. On the other hand, the PedsQL questionnaire, being composed of a considerable number of questions, has been divided into multiple views, corresponding to the 4 subgroups of questions related to different areas (health and physical activity, state of mind, relationship with others, and study or work).

**Figure 5:**
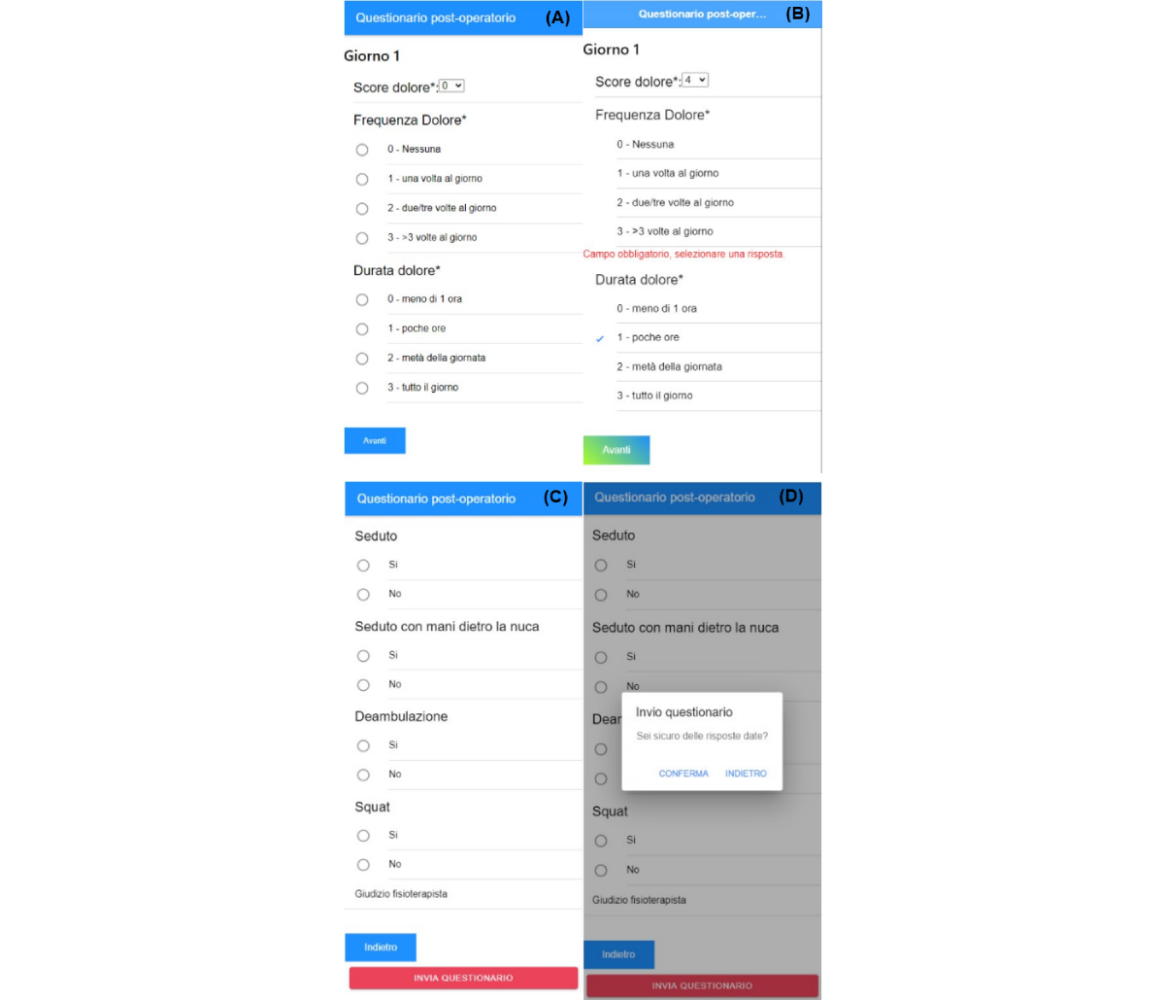
Questionnaire screen on an iOS device. (A) The first screen of the postoperative questionnaire on day 1, where the pain score can be assigned via drop-down menus, while frequency and duration are measured via multiple-choice questions. All selectable options are borrowed from the corresponding paper questionnaires. (B) If the patient, by mistake or forgetfulness, does not fill in a mandatory item, a warning appears. (C) The last screen of the postoperative questionnaire containing further questions with binary answer (yes/no) related to motor skills within the postoperative questionnaire. (D) Before the final submission of the answers, a confirmation message pops up for the user as a double-check.

Most of the questions in both kinds of paper questionnaires inherently involve a multiple-choice structure for corresponding answers. In the mobile app, this has been achieved through 2 solutions involving either a drop-down menu or multiple-option buttons, allowing the user to select at most 1 button at a time. The choice between resorting to one solution rather than another was made by taking into account the layout and display scheme of the specific screen at hand. In particular, a drop-down menu structure was preferred for pages displaying a high number of questions, as this type of configuration takes up little space. On the other hand, multiple-option buttons (eg, regarding pain duration) are particularly suitable in the case of choices among numerical values. In the developed app, the user is shown the corresponding sentence for ease of understanding and compilation, and then, the program converts the answer into the corresponding numerical value when submitting the form (Figure 5A).

For YAPFAQ and CALI9 subquestionnaires, only the overall total value is computed, sent out, and stored. On the other hand, for the PedsQL subquestionnaire, both the total for each section and the final total (by summing the values obtained from each section) are computed and saved. The motivation behind this choice lies in the fact that having the values of the individual sections at disposal could be useful to investigate the presence of an imbalance in the results between the different sections in the PedsQL, since they refer to different aspects of the patient’s daily life. All mandatory questions must be answered for the questionnaire to be correctly sent and saved. Normally, the check in a web compilation form is performed after collecting all the responses. However, in this case, since the display is divided over several successive screens, we inserted a check at the end of each display screen, so that before moving to the next page, a check is performed for the compulsory questions on that screen. If the outcome is negative, the user is prohibited from moving to the next page through an error message (Figure 5B). Finally, since the questionnaire is not editable once submitted, we have included a final security check immediately before submission. Indeed, on the last page of the questionnaire (Figure 5C), if the *submit* button is clicked, a message appears via a pop-up box in which the user is asked whether they are sure about submitting the questionnaire (Figure 5D). This further check has been added since questions contained in the last screen are not mandatory, and therefore, an unintentional click of the button could result in an inadvertent submission of the questionnaire without the possibility of revocation.

### Preliminary Analysis

We opted to select a subset of the cohort enrolled for the COPPER RCT to methodologically validate the designed framework. With the data collected from February 2022 to June 2023, we conducted an initial analysis of the results obtained in preparation for a larger study of long-term clinical markers to be conducted at the end of the enrollment.

Data collected for study inclusion included demographics, the Haller index, and presenting symptoms. All participants underwent full pulmonary and cardiology evaluations (including cardiac magnetic resonance imaging) preoperatively. Groups were established by differentiating between the pain management protocol each patient received while being an inpatient following scheduled surgery. The inclusion and exclusion criteria for the study population are indicated in Multimedia Appendix 6.

A total of 72 participants out of 88 scheduled for PE repair were analyzed, with individuals being perfectly balanced with respect to the therapy group and with respect to gender within each group (Multimedia Appendix 7). One group received standard-of-care epidural analgesia, while the other received cryoanalgesia applied to 6 intercostal nerves on each side during surgery.

As mentioned in the Introduction, PE predominantly affects the male sex, and this was confirmed by the demographic data of the enrolled participants, with 60 out of the 72 participants enrolled (83%) being male.

Moreover, the average age for each subgroup was in line with the literature on the topic, which claimed that the severity of PE increases so much during adolescence that surgery is required.

In Table 1, we display 2 measures from the CHERRIES checklist that are useful for an objective and quantitative assessment of the web app, divided by the kind of questionnaire (basal, postoperative, and follow-up). The completion rate corresponds to the ratio of users who finished the survey and users who agreed to participate. It is a marker for attrition and can involve leaving questionnaire items blank. Conversely, the completeness rate reports the percentage of missing records out of the total amount of mandatory fields to fill in. This measure functions as a preliminary indicator of the success of this method compared to manual compilation.

**Figure 6:**
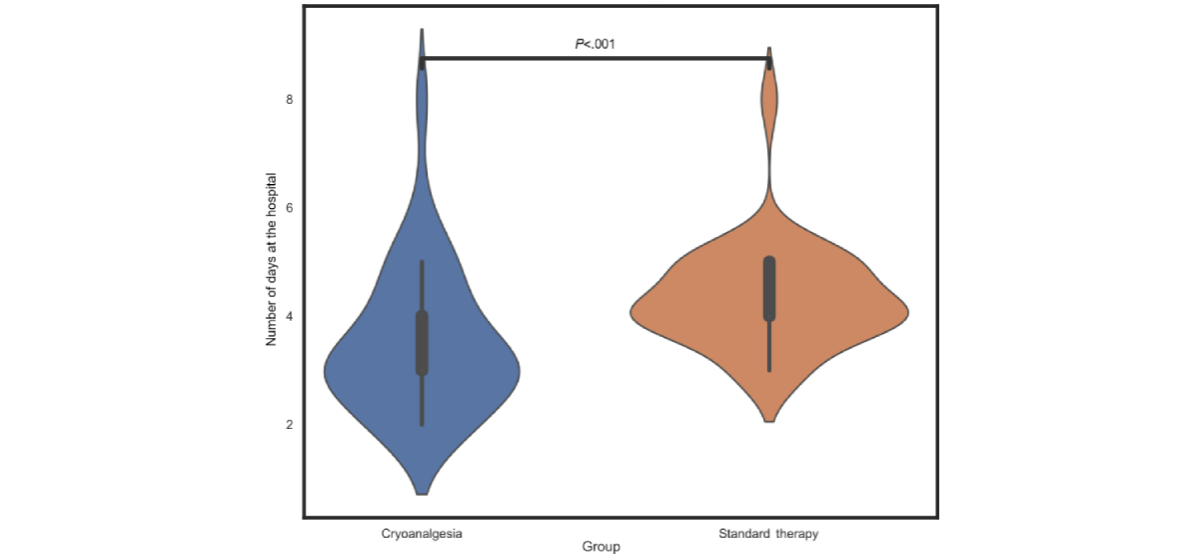
Difference in hospitalization days between the 2 treatment groups. Violin plots show the number of days at the hospital for the 2 subgroups of operated subjects. The Mann-Whitney-Wilcoxon 2-sided test with Bonferroni correction indicated that hospital stay was significantly shorter with cryoanalgesia than with standard therapy (*P*<.001; U statistic=287.5).

Leaving a detailed comparison of the measures extracted from the questionnaires for a purely clinical paper, we limited our evaluation to statistically comparing (Mann-Whitney-Wilcoxon 2-sided test with Bonferroni correction) the duration of hospital stay in the 2 groups of patients undergoing the 2 different anesthesia techniques. As shown in Figure 6, hospital stay was significantly shorter with cryoanalgesia than with standard therapy (mean 3.48, SD 1.31 days vs mean 4.32, SD 0.94 days; *P*<.001; *U* statistic=287.5). This result, which will need to be validated in an even larger cohort, is in line with the findings of previous literature [33-35].

**Table 1:** Completion and completeness rates.

Variable	Basal questionnaire			Postoperative questionnaire			Follow-up questionnaire	
	N	n (%)	N		n (%)	N		n (%)
Completion rate	72	64 (88.9)	72		54 (75.0)	72		33 (45.8)
Completeness rate	585	66 (11.3)	10,240		357 (3.5)	1692		67 (4.0)

## Discussion

### Summary

This work, which is part of the larger COPPER project, enabled the adoption of the first software app for postoperative pain management at Gaslini Hospital. This is of utmost importance given that the subjective nature of pain necessitates tools facilitating pain assessment and management in a more objective and quantifiable way. In this respect, mHealth apps have the potential to address these challenges and improve outcomes.

### Advantages of eHealth Solutions

In recent years, the use of pain-focused mHealth apps to track, assess, and manage pain has taken hold in a variety of target conditions [36-38].

These solutions best meet the demands for portable, nonintrusive, and ubiquitous solutions imposed by contemporary lifestyles to the point that resorting to digital apps for handling and gathering medical data has undisputed advantages compared with manual entry.

This shift toward the adoption of digital health approaches in community health care services allows to monitor the health of patients with higher accuracy and speed [39,40]. It reduces errors in data collection, eases the access and availability of data, and assures constant monitoring by the medical staff, leading to an improvement in the quality of care [41,42]. Moreover, the use of a digital alternative overcomes some other problems inherent to the paper solution, such as storage space and long-term retention.

Furthermore, the ease of access to web apps via a smartphone and low effort for web-based questionnaire completion make participation less demanding, increasing the patient’s involvement and contributing to positive feedback by end users.

Finally, such kinds of solutions have the potential to alleviate the burden on health care facilities by lowering the occurrence of intermediate revisits and promoting the concept of self-care among patients. Indeed, data collected through the web app are stored in a centralized database, making it easier to manage, retrieve, and analyze the collected data efficiently.

### Novelty of the Study

In the field of postoperative care of pediatric patients subjected to PE surgery, our work differs from the other few existing studies in terms of objective pain evaluation and extended daily follow-up even after hospital discharge.

In the specific case of our work, the success of adopting such a digitalized approach is evidenced by the high number of fully and correctly compiled sheets (see the Results section).

In the design of the COPPER app, we have indeed opted for maximizing patient adherence and increasing their responsibility and self-management. This engagement has been attained through constant exchange among clinicians, technicians, and end users. Specifically, the web app’s usability and user-friendliness have been tested by physicians who regularly interact with the target patient group, leveraging their expertise to suggest improvements and resolve issues.

A common challenge that impedes the long-term adoption of mHealth apps is the lack of a standardized evaluation framework for usability, accessibility, and time burden (effort required to complete questionnaires) [23]. To address this, we aimed to meet the needs of end users from the early stages by allowing them to provide feedback to health care professionals about the web app during hospitalization (initial forms were completed in the hospital) and later through the contact options provided within the app.

Moreover, the chosen UI design prioritizes making the app streamlined and intuitive, minimizing bugs, and adopting a simple and clear layout with attractive esthetics and manageable features to enhance user engagement.

To this end, we used the Bootstrap front-end framework to achieve a responsive design, ensuring optimization for devices of various sizes and resolutions, from desktops to tablets and smartphones. Bootstrap also ensures that all parts of our app follow a consistent style and design, improving visual coherence and the user experience. The UI is easy to navigate, featuring a clear menu and a summary page that displays the status of forms (completed, fillable within the current period, and to be filled in future periods). Each form takes only about 5 minutes to complete.

On the technological side, our app’s performance is guaranteed by resorting to the latest technological tools. We chose the XTENS platform due to its versatility in creating flexible data structures tailored to our needs, its user-friendly RESTful API interface, and its foundation on the Node.js runtime environment, which uses JavaScript on both the server and client sides. This setup ensures high performance, leveraging Google’s V8 engine. Additionally, XTENS provides efficient support for JSON, streamlines metadata management with PostgreSQL and Node.js, and eliminates unnecessary conversions that could impact performance.

In summary, extensibility and simple management characterizing this platform make it the optimal tool for this kind of app.

Our decision to use Angular for client development grants us the flexibility to deploy our app on various platforms. With Angular CLI, the source code can be compiled for both iOS and Android mobile devices, as well as for web-based single-page apps. Additionally, Angular offers numerous advantages, including enhanced performance, well-structured code, and robust support for mobile app development.

Moreover, resorting to Ionic exploits its main advantages as an open-source SDK for cross-platform app development, including being flexible and developer friendly, providing 1 codebase for multiple apps, comprising tools with native compatibility, offering an extensive choice of UI elements and quick prototyping, and showing testing convenience.

Finally, our app inherits all benefits inherent to hybrid apps, namely developer-side reusability of code for different platforms, time-saving in production, lowered cost of development and maintenance, availability of a web version of the mobile app, and customer-side cost saving, since the users of the hybrid mobile app do not need to spend money to acquire different mobile platforms (ie, Android, iOS, and Windows phones).

### Limitations and Future Developments

Despite their potential, pain apps inherently have several gaps and areas for improvement [23,36]. This also holds for our work, as demonstrated, for instance, by the largely improvable completion rate of follow-up questionnaires.

First, it would be beneficial to include multilanguage support, allowing the app to be translated for foreign patients. It would also be appropriate to include checks on the correctness of the format of the entered data. For example, for decimal numeric values related to clinical scores (eg, Haller index), a constraint on the use of a period instead of a comma should be inserted to ensure correct export to Excel. Similarly, it would be necessary to prevent the presence of blank records.

Engagement strategies should also take into account the target population age, thus being specifically focused on the pediatric age group. In this respect, the web app could be equipped with games or reward systems to increase motivation and incentivize questionnaire completion. In addition, to facilitate filling in on the correct date and increase the possibility of having consistent data, the app could be provided with notifications via email or instant messaging. Moreover, as it is designed and implemented, the app code allows the same structure to be maintained and used in other clinical studies that require the completion of questionnaires with minimal modifications.

The successful implementation of the proposed app has made it possible to infer an important result showing the superiority of cryoanalgesia over the standard of care in terms of length of stay. It is related to an objective measure that synthesizes numerous aspects of a patient’s postoperative course, including adverse events and pain control, which might be related to cost savings.

As a result, this digital tool will be essential for the upcoming clinical study that includes the entire cohort of patients enrolled in the COPPER clinical trial, which is devoted to a comprehensive comparison between the 2 analgesia strategies. In this respect, we plan to assess (1) the NRS score over time (from 0 to 180 days after the operation) and (2) the correlation of the difference in the PedsQL score between the 14th day of follow-up and baseline with the Haller index. It would also be interesting to include a cost-benefit analysis of the proposed protocol [34,35], as well as postoperative opioid consumption [43,44].

### Conclusions

This work enabled the development and delivery of the first web app in use at Giannina Gaslini Children’s Hospital for both web and mobile devices for the completion of questionnaires within a clinical trial. The data currently collected include (1) medical history or clinical information, (2) PE evaluations, (3) postoperative evaluations, and (4) follow-up assessments.

The emergence of such an app plays a pivotal role in empowering individuals to actively participate in managing their own health and well-being, facilitating the transformation of health and care services and shifting them toward digitalized, person-centered, and community-based models of care.

## Appendix

Multimedia Appendix 1 Postoperative questionnaire to be filled out within the first 13 days after the surgical intervention.

Multimedia Appendix 2 Youth Acute Pain Function Ability Questionnaire (YAPFAQ) assessment scale included both in the postoperative and follow-up surveys.

Multimedia Appendix 3 Follow-up questionnaire to be filled out from the 14th day until 6 months after the surgical intervention.

Multimedia Appendix 4 Pediatric Quality of Life Inventory (PedsQL) assessment scale (specific of the only follow-up form), with raw scores not yet transformed into the range 0-100.

Multimedia Appendix 5 Checklist for Reporting Results of Internet E-Surveys (CHERRIES).

Multimedia Appendix 6 Eligibility criteria for the Cryoanalgesia for Pain Management After Pectus Excavatum Repair (COPPER) study involving patients with pectus excavatum.

Multimedia Appendix 7 Population demographics. Bar plots represent the distribution of the population under examination in the 2 treatment groups, with further division by gender. To complement this, the mean age and relative SD are shown for each subgroup.

## Abbreviations

API: application programming interface
CALI9: Child Activity Limitations Interview-9
CHERRIES: Checklist for Reporting Results of Internet E-Surveys
COPPER: Cryoanalgesia for Pain Management After Pectus Excavatum Repair
NRS: Numeric Pain Rating Scale
PE: pectus excavatum
PedsQL: Pediatric Quality of Life Inventory
RCT: randomized controlled trial
SDK: software development kit
UI: user interface
YAPFAQ: Youth Acute Pain Function Ability Questionnaire

## Data Availability

The data sets generated during or analyzed during this study are not publicly available due to privacy or ethical restrictions but are available from the corresponding author upon reasonable request. The code of the app is available at [45].
