# Supplementary material for: Comparison of Analgesia Methods Through a Web Platform in Patients Undergoing Thoracic Surgery: Pilot Design, Implementation, and Validation Study
Source: JMIR Form Res. 2024 Oct 8;8:e56674. doi: 10.2196/56674 (PMC11496914; doi:10.2196/56674)
Supplement: Multimedia Appendix 1 [file formative_v8i1e56674_app1.docx]

**Multimedia Appendix 1.** Postoperative questionnaire to be filled out within the first 13 days after the surgical intervention.

This paper survey has been used as an outline for the development of its digital counterpart delivered through our application.

**
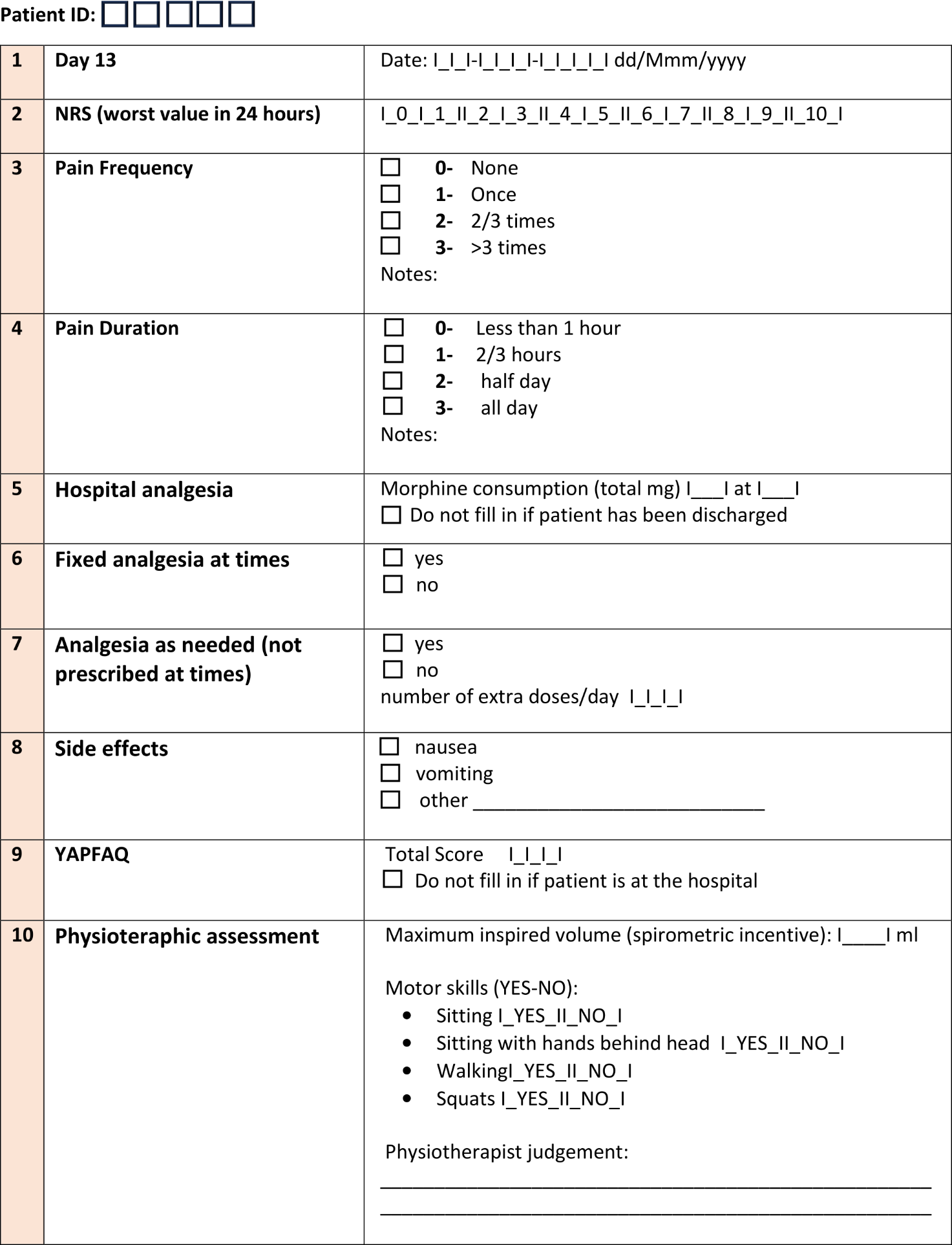
**
