# Supplementary material for: Comparison of Analgesia Methods Through a Web Platform in Patients Undergoing Thoracic Surgery: Pilot Design, Implementation, and Validation Study
Source: JMIR Form Res. 2024 Oct 8;8:e56674. doi: 10.2196/56674 (PMC11496914; doi:10.2196/56674)

**Multimedia Appendix 2.** Youth Acute Pain Function Ability Questionnaire (YAPFAQ) assessment scale included both in the postoperative and follow-up surveys.


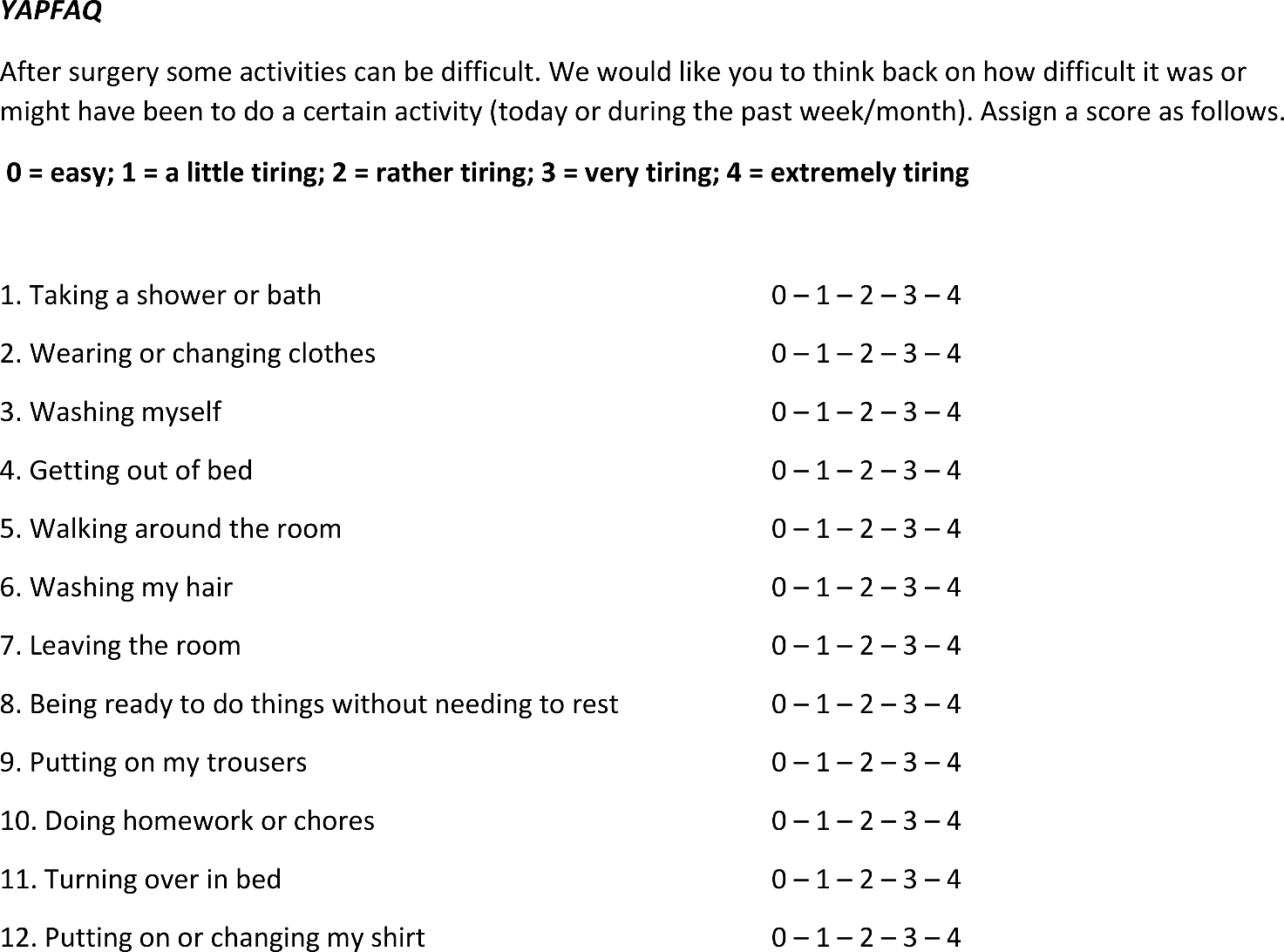

Supplement: Multimedia Appendix 2 [file formative_v8i1e56674_app2.docx]
