# Supplementary material for: Comparison of Analgesia Methods Through a Web Platform in Patients Undergoing Thoracic Surgery: Pilot Design, Implementation, and Validation Study
Source: JMIR Form Res. 2024 Oct 8;8:e56674. doi: 10.2196/56674 (PMC11496914; doi:10.2196/56674)

**Multimedia Appendix 3.** Follow-up questionnaire to be filled out from the 14th day until 6 months after the surgical intervention.

This paper survey has been used as an outline for the development of its digital counterpart delivered through our application.


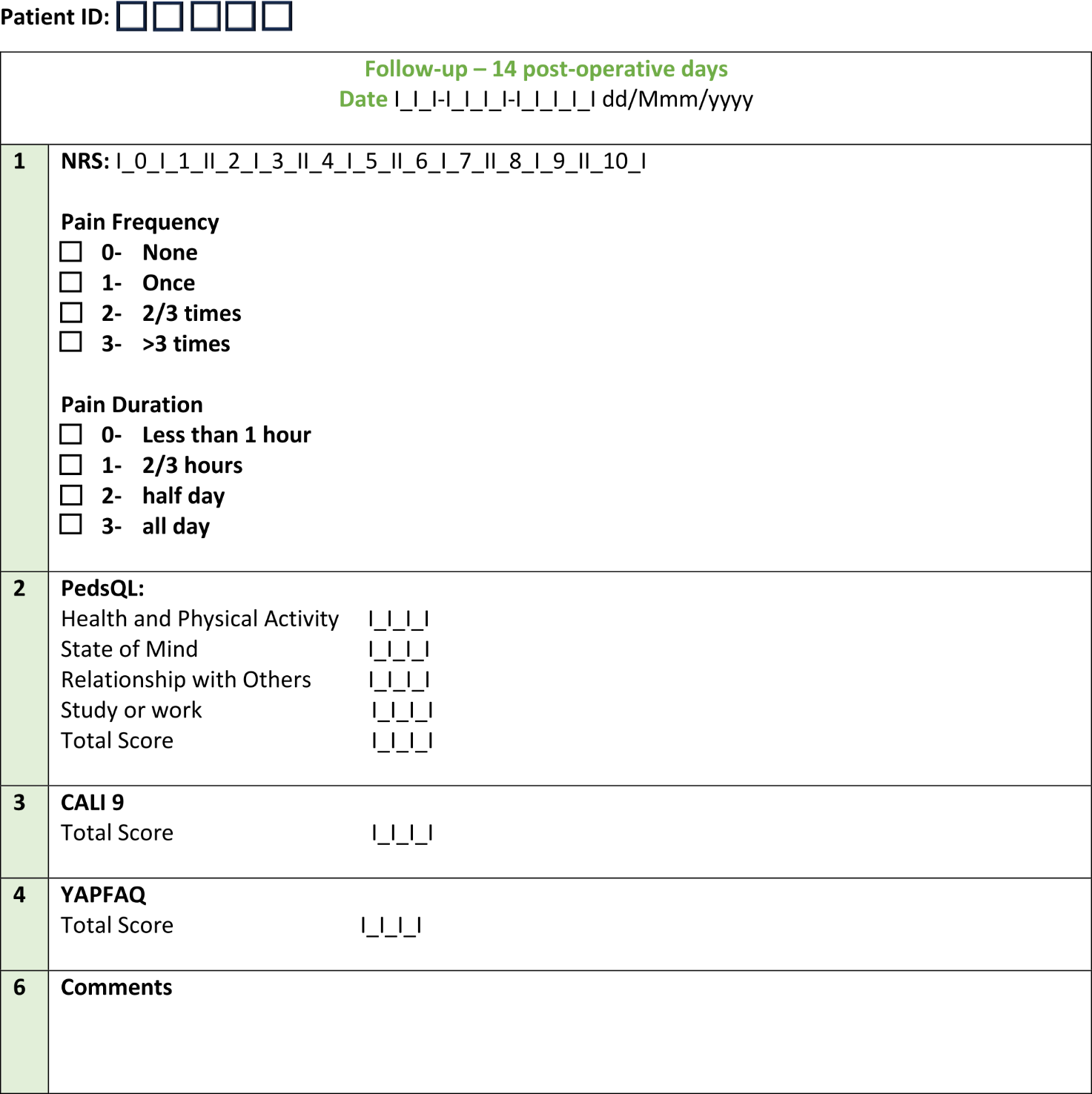

Supplement: Multimedia Appendix 3 [file formative_v8i1e56674_app3.docx]
