# Supplementary material for: Comparison of Analgesia Methods Through a Web Platform in Patients Undergoing Thoracic Surgery: Pilot Design, Implementation, and Validation Study
Source: JMIR Form Res. 2024 Oct 8;8:e56674. doi: 10.2196/56674 (PMC11496914; doi:10.2196/56674)

**Multimedia Appendix 4.** Pediatric Quality of Life Inventory (PedsQL) assessment scale (specific of the only follow-up form), with raw scores not yet transformed into the range 0-100.


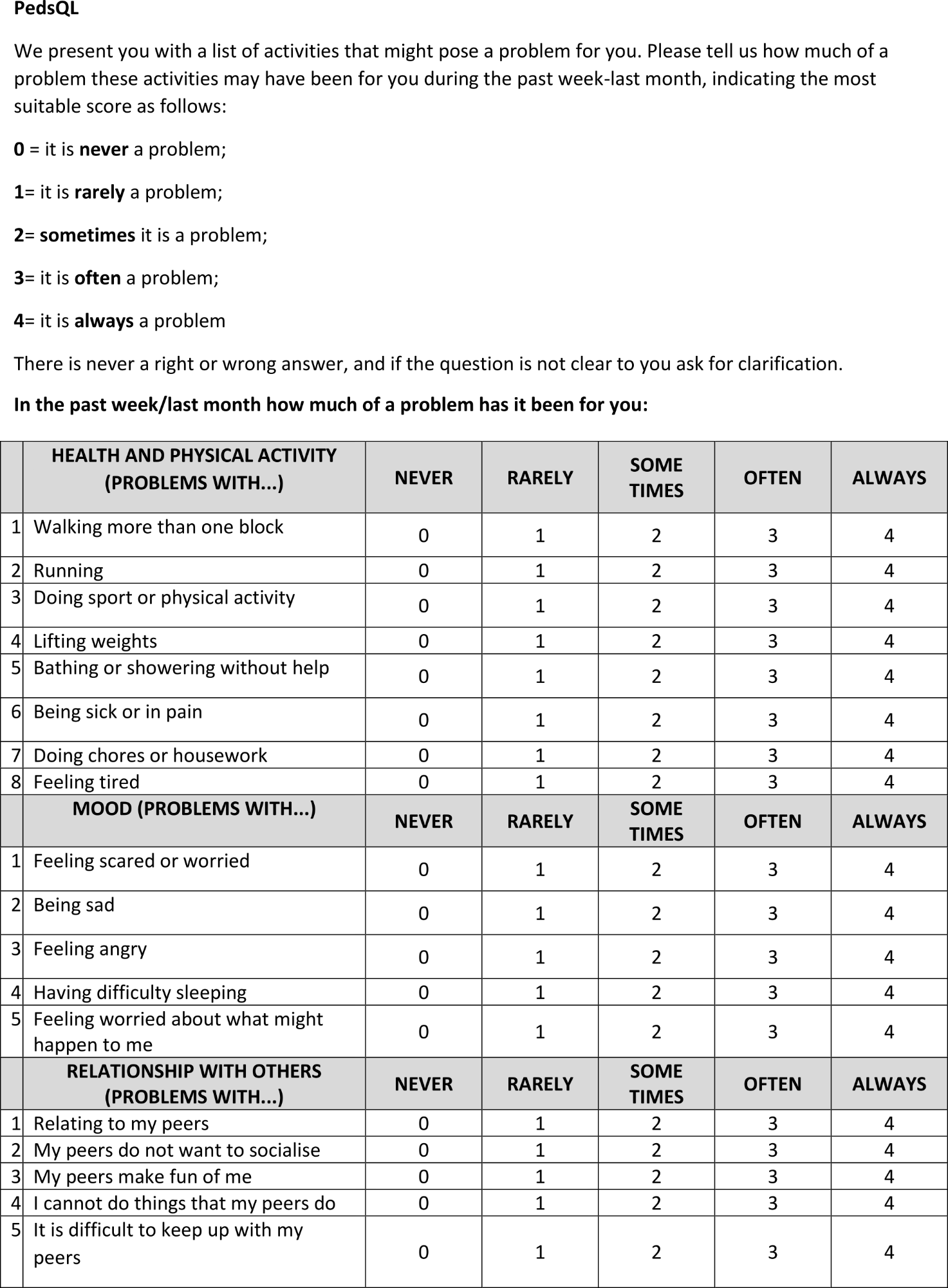

Supplement: Multimedia Appendix 4 [file formative_v8i1e56674_app4.docx]
