# Supplementary material for: Comparison of Analgesia Methods Through a Web Platform in Patients Undergoing Thoracic Surgery: Pilot Design, Implementation, and Validation Study
Source: JMIR Form Res. 2024 Oct 8;8:e56674. doi: 10.2196/56674 (PMC11496914; doi:10.2196/56674)
Supplement: Multimedia Appendix 5 [file formative_v8i1e56674_app5.docx]

**Multimedia Appendix 5.** Checklist for Reporting Results of Internet E-Surveys (CHERRIES).

| \| 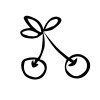 \| **Checklist for Reporting Results of Internet E-Surveys (CHERRIES)** \| \| --- \| --- \| | | | |
| --- | --- | --- | --- | --- | --- |
| ***Item Category*** | ***Checklist Item*** | ***Explanation*** |  |
| **Design** |  |  |  |
|  | Describe survey design | Describe target population, sample frame. Is the sample a convenience sample? (In “open” surveys this is most likely.) | Randomized active controlled, parallel group, single-centre, trial (category IIb medical device). The sample size is based on the assumption that the overall score of the primary outcome measure PedsQL shows a mean improvement of 20 points [SD = 30]. To test the null hypothesis of equality of treatment at α = .05 with 80% power and assuming a uniform dropout rate of 10%, 44 patients in each group would be sufficient. |
| **IRB (Institutional Review Board) approval and informed consent process** |  |  |  |
|  | IRB approval | Mention whether the study has been approved by an IRB. | The study has been approved by IRB number 278/2021 – DB id 11421 |
|  | Informed consent | Describe the informed consent process. Where were the participants told the length of time of the survey, which data were stored and where and for how long, who the investigator was, and the purpose of the study? | The participants have been asked for informed consent the day before hospital admission. They were told the length of time of each survey (5 minutes per form). The investigators were named. The purpose of the study and all items in the form were explained the day before hospital admission. |
|  | Data protection | If any personal information was collected or stored, describe what mechanisms were used to protect unauthorized access. | Both web app and XTENS web application are password protected and all data transmission is done via SSL encryption. Only the web app can access the XTENS API due to IP check. |
| **Development and pre-testing** |  |  |  |
|  | Development and testing | State how the survey was developed, including whether the usability and technical functionality of the electronic questionnaire had been tested before fielding the questionnaire. | The surveys were developed internally at the Gaslini Institute. All tests were conducted by physicians who typically work with this specific patient group, providing them with the expertise to recommend improvements and address any issues. |
| **Recruitment process and description of the sample having access to the questionnaire** |  |  |  |
|  | Open survey versus closed survey | An “open survey” is a survey open for each visitor of a site, while a closed survey is only open to a sample which the investigator knows (password-protected survey). | It is a closed survey: all participants have been selected by investigators |
|  | Contact mode | Indicate whether or not the initial contact with the potential participants was made on the Internet. (Investigators may also send out questionnaires by mail and allow for Web-based data entry.) | All participants are patients at Gaslini Institute and have been contacted orally at the time of visit. They have been selected and added as users on XTENS web platform. |
|  | Advertising the survey | How/where was the survey announced or advertised? Some examples are offline media (newspapers), or online (mailing lists – If yes, which ones?) or banner ads (Where were these banner ads posted and what did they look like?). It is important to know the wording of the announcement as it will heavily influence who chooses to participate. Ideally the survey announcement should be published as an appendix. | N/A  No advertisement since it is about a closed survey |
| **Survey administration** |  |  |  |
|  | Web/E-mail | State the type of e-survey (eg, one posted on a Web site, or one sent out through e-mail). If it is an e-mail survey, were the responses entered manually into a database, or was there an automatic method for capturing responses? | It is a web site e-survey. Data are entered by participants directly via app and answers are stored in XTENS web application database |
|  | Context | Describe the Web site (for mailing list/newsgroup) in which the survey was posted. What is the Web site about, who is visiting it, what are visitors normally looking for? Discuss to what degree the content of the Web site could pre-select the sample or influence the results. For example, a survey about vaccination on a anti-immunization Web site will have different results from a Web survey conducted on a government Web site | Both web app and admin web application can be accessed by enabled users with correct credentials. Web app only stores data; no filter or selection has been made in any step of storing process |
|  | Mandatory/voluntary | Was it a mandatory survey to be filled in by every visitor who wanted to enter the Web site, or was it a voluntary survey? | It was a voluntary survey |
|  | Incentives | Were any incentives offered (eg, monetary, prizes, or non-monetary incentives such as an offer to provide the survey results)? | No |
|  | Time/Date | In what timeframe were the data collected? | Data has been collected between February 2022 to June 2023 |
|  | Randomization of items or questionnaires | To prevent biases items can be randomized or alternated. | Due to the nature of the survey, randomization of items was not performed.  However, items could be randomized, the results would not change. |
|  | Adaptive questioning | Use adaptive questioning (certain items, or only conditionally displayed based on responses to other items) to reduce number and complexity of the questions. | Where possible, we have used adaptive questioning; i.e. some questions are Yes/No, and only if answer is yes we ask for specification |
|  | Number of Items | What was the number of questionnaire items per page? The number of items is an important factor for the completion rate. | We tried to divide the survey in several pages to make them more readable. Maximum number of items per page is 12 (YAPFAQ questionnaire). |
|  | Number of screens (pages) | Over how many pages was the questionnaire distributed? The number of pages is an important factor for the completion rate. | We have 2 types of survey: post operation survey has 4 pages, instead follow up survey has 8 pages with PedSQL questionnaire divided in 4 pages |
|  | Completeness check | It is technically possible to do consistency or completeness checks before the questionnaire is submitted. Was this done, and if “yes”, how (usually JAVAScript)? An alternative is to check for completeness after the questionnaire has been submitted (and highlight mandatory items). If this has been done, it should be reported. All items should provide a non-response option such as “not applicable” or “rather not say”, and selection of one response option should be enforced. | Mandatory questions are highlighted if not answered. Several questions do not have “not applicable” answer but questions are time-related to the day of survey completion |
|  | Review step | State whether respondents were able to review and change their answers (eg, through a Back button or a Review step which displays a summary of the responses and asks the respondents if they are correct). | Participants can change their answers and for any page there is a “back” button. After confirming the survey, users are no more able to change their answers. They must contact administrators who can change answers by XTENS web application |
| **Response rates** |  |  |  |
|  | Unique site visitor | If you provide view rates or participation rates, you need to define how you determined a unique visitor. There are different techniques available, based on IP addresses or cookies or both. | N/A  The only visitors are the recruited patients |
|  | View rate (Ratio of unique survey visitors/unique site visitors) | Requires counting unique visitors to the first page of the survey, divided by the number of unique site visitors (not page views!). It is not unusual to have view rates of less than 0.1 % if the survey is voluntary. | N/A  As this was a targeted questionnaire for specific patients, information such as the view rate was not relevant |
|  | Participation rate (Ratio of unique visitors who agreed to participate/unique first survey page visitors) | Count the unique number of people who filled in the first survey page (or agreed to participate, for example by checking a checkbox), divided by visitors who visit the first page of the survey (or the informed consents page, if present). This can also be called “recruitment” rate. | N/A  As this was a targeted questionnaire for specific patients, information such as the participation rate was not relevant. |
|  | Completion rate (Ratio of users who finished the survey/users who agreed to participate) | The number of people submitting the last questionnaire page, divided by the number of people who agreed to participate (or submitted the first survey page). This is only relevant if there is a separate “informed consent” page or if the survey goes over several pages. This is a measure for attrition. Note that “completion” can involve leaving questionnaire items blank. This is not a measure for how completely questionnaires were filled in. (If you need a measure for this, use the word “completeness rate”.) | - 88% (64 out of 72) of patients have completed baseline questionnaire - 75% (54 out of 72) of patients have filled out at least one post-operative questionnaire - 46% (33 out of 72) of patients have filled out at least one follow-up questionnaire |
| **Preventing multiple entries from the same individual** |  |  |  |
|  | Cookies used | Indicate whether cookies were used to assign a unique user identifier to each client computer. If so, mention the page on which the cookie was set and read, and how long the cookie was valid. Were duplicate entries avoided by preventing users access to the survey twice; or were duplicate database entries having the same user ID eliminated before analysis? In the latter case, which entries were kept for analysis (eg, the first entry or the most recent)? | Cookies have been used to assign a unique user identifier to each client computer at the login page after logging in. |
|  | IP check | Indicate whether the IP address of the client computer was used to identify potential duplicate entries from the same user. If so, mention the period of time for which no two entries from the same IP address were allowed (eg, 24 hours). Were duplicate entries avoided by preventing users with the same IP address access to the survey twice; or were duplicate database entries having the same IP address within a given period of time eliminated before analysis? If the latter, which entries were kept for analysis (eg, the first entry or the most recent)? | No IP check has been done |
|  | Log file analysis | Indicate whether other techniques to analyze the log file for identification of multiple entries were used. If so, please describe. | We have only database log file, but multiple entries cannot be entered. |
|  | Registration | In “closed” (non-open) surveys, users need to login first and it is easier to prevent duplicate entries from the same user. Describe how this was done. For example, was the survey never displayed a second time once the user had filled it in, or was the username stored together with the survey results and later eliminated? If the latter, which entries were kept for analysis (eg, the first entry or the most recent)? | It is a closed survey, after login the app manages what the user can enter. If a survey is already confirmed, users cannot open it again |
| **Analysis** |  |  |  |
|  | Handling of incomplete questionnaires | Were only completed questionnaires analyzed? Were questionnaires which terminated early (where, for example, users did not go through all questionnaire pages) also analyzed? | Only completed and confirmed survey were analyzed |
|  | Questionnaires submitted with an atypical timestamp | Some investigators may measure the time people needed to fill in a questionnaire and exclude questionnaires that were submitted too soon. Specify the timeframe that was used as a cut-off point, and describe how this point was determined. | N/A |
|  | Statistical correction | Indicate whether any methods such as weighting of items or propensity scores have been used to adjust for the non-representative sample; if so, please describe the methods. | N/A |

Adapted from: Eysenbach G. Improving the quality of Web surveys: the Checklist for Reporting Results of Internet E-Surveys (CHERRIES). J Med Internet Res. 2004;6(3):e34. doi: 10.2196/jmir.6.3.e34. PMID: 15471760. PMCID: PMC1550605. Available from: https:// www.jmir.org/2004/3/e34/. © Gunther Eysenbach. Originally published in the Journal of Medical Internet Research (http://www.jmir.org), 29.9.2004. Creative Commons Attribution License (http:// www.creativecommons.org/licenses/by/2.0/). N/A: Not Applicable
