# Supplementary material for: Comparison of Analgesia Methods Through a Web Platform in Patients Undergoing Thoracic Surgery: Pilot Design, Implementation, and Validation Study
Source: JMIR Form Res. 2024 Oct 8;8:e56674. doi: 10.2196/56674 (PMC11496914; doi:10.2196/56674)
Supplement: Multimedia Appendix 6 [file formative_v8i1e56674_app6.docx]

**Multimedia Appendix 6. Eligibility criteria for the** Cryoanalgesia for Pain Management After Pectus Excavatum Repair **(COPPER) study involving patients with pectus excavatum.**

| Inclusion Criteria | Exclusion Criteria |
| --- | --- |
| - Patients undergoing pectus excavatum repair with NUSS technique - aged 12 years or above - informed consent signed for cryoanalgesia | - Age of 11 years or below - Refuse to receive cryoanalgesia or epidural catheter as primary pain relief - Any contraindication to cryoanalgesia - Difficult follow-up for geographical reasons and/or impossibility by the patient to understand how to perform self-measurements |
